# Automated Epileptic Seizure Detection in Pediatric Subjects of CHB-MIT EEG Database—A Survey

**DOI:** 10.3390/jpm11101028

**Published:** 2021-10-15

**Authors:** J. Prasanna, M. S. P. Subathra, Mazin Abed Mohammed, Robertas Damaševičius, Nanjappan Jothiraj Sairamya, S. Thomas George

**Affiliations:** 1 Department of Electronics and Instrumentation Engineering, Karunya Institute of Technology and Sciences, Coimbatore 641114, India; prasu1796@gmail.com (J.P.); sairamyanj@karunya.edu.in (N.J.S.); 2 Department of Robotics Engineering, Karunya Institute of Technology and Sciences, Coimbatore 641114, India; subathra@karunya.edu; 3 Information Systems Department, College of Computer Science and Information Technology, University of Anbar, Ramadi 31000, Anbar, Iraq; mazinalshujeary@uoanbar.edu.iq; 4 Department of Applied Informatics, Vytautas Magnus University, 44404 Kaunas, Lithuania; 5 Faculty of Applied Mathematics, Silesian University of Technology, 44-100 Gliwice, Poland; 6 Department of Biomedical Engineering, Karunya Institute of Technology and Sciences, Coimbatore 641114, India

**Keywords:** epilepsy, electroencephalogram, EEG, seizure detection, CHB-MIT database, feature extraction, classification

## Abstract

Epilepsy is a neurological disorder of the brain that causes frequent occurrence of seizures. Electroencephalography (EEG) is a tool that assists neurologists in detecting epileptic seizures caused by an unexpected flow of electrical activities in the brain. Automated detection of an epileptic seizure is a crucial task in diagnosing epilepsy which overcomes the drawback of a visual diagnosis. The dataset analyzed in this article, collected from Children’s Hospital Boston (CHB) and the Massachusetts Institute of Technology (MIT), contains long-term EEG records from 24 pediatric patients. This review paper focuses on various patient-dependent and patient-independent personalized medicine approaches involved in the computer-aided diagnosis of epileptic seizures in pediatric subjects by analyzing EEG signals, thus summarizing the existing body of knowledge and opening up an enormous research area for biomedical engineers. This review paper focuses on the features of four domains, such as time, frequency, time-frequency, and nonlinear features, extracted from the EEG records, which were fed into several classifiers to classify between seizure and non-seizure EEG signals. Performance metrics such as classification accuracy, sensitivity, and specificity were examined, and challenges in automatic seizure detection using the CHB-MIT database were addressed.

## 1. Introduction

According to the World Health Organization (WHO), approximately 50 million people in the world are affected by epilepsy [1]. In the global population, about 180,000 new cases of epilepsy are recorded each year [2], while nearly three quarters of epilepsy patients do not have access to medical treatment. Epilepsy is a neurological disease of the brain [3] in which seizures frequently occur due to an unpredicted stream of electrical motion, which causes the abnormal consequences of extreme and hypersynchronous action of neurons in the brain. Due to the frequent occurrence of seizures, an epileptic patient may experience unconsciousness and amnesia, mild depression, persistent headache. It causes body movement disorders and even death [4]. In the population affected by epileptic seizures, about 70% are adults and 30% are children. Epileptic seizures are caused by low oxygen levels during birth and head injuries that ensued during pregnancy, brain tumors, and abnormal levels of sodium or blood sugar. In about 70% of the cases, the cause of epilepsy in adults and children is not discovered. Seizures are classified into partial (focal) and generalized [5], where some part of the cerebral hemisphere is affected in focal seizures, and the whole brain is affected in generalized epileptic seizures. Some types of generalized seizures are tonic-clonic or convulsive seizures, absence seizures, atonic seizures, clonic seizures, tonic seizures, and myoclonic seizures [6]. Based on the progress of the event, epilepsy is classified into four stages, namely interictal, preictal, ictal, and postictal. The occurrence of epileptic seizures is referred to as the ictal stage, the timelapse of around 1–15 min before the incidence of a seizure is referred to as the preictal stage, and the stage after the occurrence of the seizure is referred to as the postictal stage. The time interval between the two seizures is considered an interictal stage [7].

Electroencephalography (EEG) is a noninvasive tool that is useful for the extraction of information about the electrical activity of the brain that indicates a very large number of neuronal membrane potentials that will be measured by placing electrodes on the scalp, which plays a vital role in diagnoses of epilepsy. Visual diagnosis of epileptic seizures using an EEG record is a monotonous task and consumes tremendous time for the neurologist. On the other hand, the EEG signal contains a potent biomarker to recognize various abnormal brain conditions, including depression [8] and seizures [9]. Therefore, it is necessary to automate the detection of epilepsy by recognizing the abnormal EEG condition by employing machine learning approaches [10] to achieve the goals of personalized medicine.

Personalized medicine, also known as precision medicine, is a medical concept in which people are divided into groups, and medical decisions, procedures, and/or drugs are personalized to the individual patient based on their expected response or risk of disease. EEG signals are a useful tool in precision medicine and personalized medicine. Automated diagnosis of epilepsy is a focus area for researchers that seek to reduce time consumption and computational cost. It consists primarily of two parts, such as feature extraction using various digital signal processing (DSP) methods and operators, to compute relevant features and classification stage to discriminate healthy (normal) and abnormal EEG signals or EEG signals corresponding to different mental states of the subject [11,12,13].

In previous work, the authors proposed a machine learning method for the classification of seizures using scalp EEG and a support vector machine (SVM) classifier, which achieved an accuracy of 90% [14]. A wavelet-based feature extraction technique was performed to extract the statistical feature of the mean absolute deviation (MAD). The extracted features were fed into the linear discriminant analysis classifier (LDA) to differentiate epileptic and non-epileptic events and attained an accuracy of 96.5% [15]. The continuous wavelet transform (CWT) was developed to extract characteristics, and the SVM classifier was adapted to perform epilepsy classification, which achieved a sensitivity of 52.2% in [16]. A data-driven approach was involved, and a fourth-order FIR filter was used to give 256 features that were nourished into the SVM classifier to discriminate between normal and abnormal EEG records in [17]. The patient-specific seizure detection approach was demonstrated by supervised low-power sensor nodes for efficient sensing, and the spectral features were extracted and fed to SVM, which acquired the sensitivity, latency, and false alarm of 94.70%, 5.83 s, and 0.199 per hour, respectively, in [18].

A discrete wavelet transform (DWT) was employed to decompose EEG signals in [19]. Energy and a normalized coefficient of variance were measured from each coefficient and fed into the LDA classifier to identify seizure epochs, which achieved a precision of 91.8%, sensitivity of 83.6%, and specificity of 100%. An energy efficient filter architecture was developed using distributed quad-LUT, and a linear SVM classifier was used to classify epileptic and non-epileptic signals, which achieved an accuracy of 82.7% with a latency of 2 s [20].

Conditional mutual information maximization (CMIM) as a feature selection method was introduced to select features from the extracted time, frequency, time-frequency, and nonlinear features. The extracted features were fed into the SVM classifier to discriminate the EEG signals and obtained 90.62% sensitivity and 99.05% specificity [21]. Binary classifiers for a patient-specific classification were implemented, resulting in a sensitivity of 89% and a specificity of 93% [22].

Wavelet-based nonlinear features were extracted, which was used for the classification process using an Extreme Learning Machine (ELM), which gave the sensitivity of 92.6% and a false detection rate of 0.078 [23]. Wavelet transform (WT) was applied to decompose the signals, and wavelet-based features were extracted, which were fed into the linear classifier, and achieved a sensitivity of 98.5% with a latency of 1.76 s [24].

A patient-specific seizure detector based on unsupervised feature learning, namely stacked autoencoders, was used to learn features from raw EEG signals in [25]. The extracted features were fed to the logistic classifier for the discrimination of EEG signals.

Recurrence quantification analysis (RQA) was developed to detect epileptic seizures, and the signal-to-noise ratio (SNR) was calculated by applying a wavelet and notch filter, which obtained 97.4% sensitivity and 93.5% specificity [26]. A fuzzy entropy-based approach with SVM was used to classify EEG signals, which attained precision of 98.31%, specificity of 98.36%, and sensitivity of 98.27% [27]. An automatic mobile-based approach for seizure detection was proposed by analyzing EEG signals in the time domain, frequency domain, and time-frequency domain. From the analyzed signals, several characteristics were calculated, and the sequential forward feature selection method was used to select informative characteristics, which were fed into k-means clustering for classification [28]. The feature extraction approach of EEG signals mapped in the two-dimensional space was proposed, and several classifiers were adopted, which achieved a sensitivity of 70.19% and a specificity of 97.74% [29].

The multitask learning method was applied to the long data record in which the challenges related to variation between patients and intrapatients were resolved by training an SVM classifier to distinguish epileptic and non-epileptic signals [30]. RQA was used to characterize the EEG signal, and the extracted features were fed into the error-correcting output code (ECOC) classifier, which acquired a sensitivity of 97.4% and a specificity of 93.5%, respectively [31]. The supervised machine learning method for the classification of seizures was introduced using scalp EEG and the magnetic resonance imaging approach to obtain a sensitivity of 93% and a specificity of 94% using the K-Nearest Neighbor classifier (K-NN) [32]. The Singular Lorenz Measures Method (SLMM) has been proposed for feature extraction where the decomposition of the EEG record is performed by applying DWT, and the extracted features based on SLMM were delivered to different classifiers to provide efficient classification that refined the detection accuracy of 90% [33].

The patient-specific method of the Poincare section, LDA, and Naïve Bayesian (NB) classifiers was used, which attained a sensitivity of 88.27% [34]. A single-channel automatic seizure detection algorithm was developed based on a statistical approach performed by filtering, peak-to-peak rectification, smoothing, semi-logarithmic compression, and time compression, which achieved 88.50% sensitivity with a false detection rate of 0.18 [35]. Classical characteristics and singular values such as average power, delta band average power, variance, and mean were extracted by applying the singular value decomposition (SVD) technique, and the SVM classifier was used for seizure classification, which achieved an average precision of 94.82% [36]. The frequency division multiplexing filter and dual detector architecture were implemented to detect 16 channel seizure events, and the SVM classifier was used to provide a high sensitivity of 95.7% [37].

Multidimensional parallel factor analysis (PARAFAC) was used to extract spatial spectral characteristics, and the adaptive zero training technique was proposed with the intention of better classification when the LDA and SVM classifier was adopted [38]. The context learning model was intended to detect epileptic seizures by extracting the hidden inherent features with a sparse autoencoder. Hidden and temporal features were given to the binary classifier, which achieved an error rate of 22.93% [39]. A supervised machine learning method, namely principal component analysis (PCA) and LDA, was introduced with a k-NN classifier to classify EEG signals using the characteristics extracted from the decomposed wavelets, which achieved a sensitivity and specificity of 88% [40].

The stationary wavelet transform (SWT) was applied for seizure detection based on a nonspecific patient procedure with the LDA classifier for accurate classification, which achieved 99.9% specificity and 87.5% sensitivity [41]. Interpolated histogram features (IHF) were extracted from the EEG signal, and to select informative features, a Bayesian classifier and a Hunting search algorithm were used in offline seizure detection. A multilayer perceptron (MLP) classifier was trained with the optimal selected features for online seizure detection, which achieved an accuracy of 86.56% [42].

A patient-specific seizure detection algorithm was developed using SVM and linear SVM and achieved high sensitivity and specificity of 95.1% and 96.2%, respectively [43]. EEG classification based on a multichannel machine learning approach in a wearable environment was implemented by on-chip classification where the features were extracted and given to the linear SVM classifier. The nonlinear classifier was applied and found that the sensitivity and specificity of the nonlinear SVM were refined at a rate of 12.4% and 3.56% compared to those of the Linear SVM [44]. A robust learning framework was proposed to alleviate the class imbalance in the CHB-MIT dataset for seizure detection. It adopts RUSBoost, which increases the performance of the classifier [45].

Multilevel wavelet decomposition was adapted to extract features based on magnitude and spectral energy variation, and fed into SVM and ELM classifiers, thereby achieving a sensitivity of 99.48% [46]. The MLP-based neural network was used to detect epileptic seizures by training a classifier based on the backpropagation algorithm [47]. The patient-independent and patient-dependent classification was developed by investigating wavelet characteristics with an SVM classifier, achieving an overall precision of 96.87% [48]. Temporal and spectral characteristics were extracted using WT, and these characteristics were given to ELM for automated epilepsy classification, which achieved 94.85% classification accuracy [49]. The low-complexity seizure prediction technique was explored for the use of attractor state analysis where the linear spectral characteristic was evaluated, resulting in a sensitivity of 86.67% [50]. SVD was applied, and eigenvalues were calculated to detect seizures [51].

The phase locking value was analyzed for the prediction of seizures using empirical mode decomposition (EMD), and other types of EMD were proposed. The extracted features were fed into the SVM classifier to perform the classification [52]. An unsupervised method of predicting seizures was used to perform a classification with the mallet scattering transform to analyze an EEG signal, which attained a specificity of 98% and a sensitivity of 78% [53]. A multivariate method of empirical wavelet transform (EWT) was performed to extract the characteristics, and different classifiers were used for classification, which achieved a sensitivity of 97.91%, specificity of 99.41%, and precision of 99.41% [54].

Supervised detection of epileptic seizures was proposed using the local Gabor binary pattern (LGBP) method, and features were extracted using sparse rational decomposition. These characteristics were nourished in different classifiers and achieved a net sensitivity of 70.4% [55]. An energy-based seizure detection algorithm was performed, and the genetic algorithm for optimization was used to refine the accuracy of the detection [56]. The Fast Wavelet Decomposition (FWD) approach was applied to extract features that were given to the Relevance Vector Machine (RVM) for the discrimination of epileptic and non-epileptic signals achieving a sensitivity of 96% [57]. The logarithm of the variance of detail obtained by single wavelet-based features was proposed to perform the patient-dependent epileptic seizure classification using 4-fold cross-validation to categorize seizure and non-seizure activity, which achieved an accuracy, sensitivity, and specificity of 93.24%, 83.34%, and 93.53% [58]. Feature extraction was performed by segmenting the EEG signal based on coinciding change points to study the quasi-stationary nature of EEG for prediction of epileptic seizures [59]. The sparse feature selection procedure was used to extract eight different sub-bands of spectral power features that were selected, and a kernel sparse representation classifier was used to predict epileptic seizures, which achieves a sensitivity of 86.11% [60].

The Field Programmable Gate Array (FPGA) approach was implemented for automatic seizure detection to examine the amplitude and frequency components. The timing of seizure detection was 1.56 ns and 7.572 ns, respectively [61]. The slope-based detection (SBD) accelerator was experimented with to detect real-time seizures and achieved 100% sensitivity with 0.5 s latency [62]. A PCA introduced using a distance-based change point detector provided a sensitivity rate of 87% [63]. A fuzzy rule-based and layered directed acyclic graph SVM (LDAG-SVM) was developed accordingly, reaching an accuracy of 98% and a sensitivity of 99% [64]. The convolutional neural network (CNN) approach was developed to interpret seizures and non-seizures, which achieved a sensitivity of 81.4% [65]. The WT methods were applied to analyze the EEG signals, and time-frequency-based features were extracted.

The extracted features were fed into a fuzzy classifier for discrimination of epileptic and non-epileptic EEG signals, which attained an accuracy of 96.48% [66]. The high-dimensional phase space through the Poincare section and two classifiers, such as the SVM classifier and the NB classifier, was analyzed to attain an accuracy of 96.77% [67]. EMD, DWT, and wavelet packet decomposition (WPD) methods were applied to characterize the EEG signals. Statistical characteristics were extracted for automatic detection of seizures, which achieved an overall accuracy of 100% [68]. Long short-term memory (LSTM) networks were adopted for the prediction of epileptic seizures by enlarging deep learning algorithms with a CNN [69]. The unsupervised method based on a four-segment selection-based method for the detection of seizures was used and achieved a sensitivity of 89% [70]. Prediction accuracy of 90.5% was achieved by employing a deep CNN [71]. Adopting a lightweight VGGNet approach for seizure detection reached better accuracy, sensitivity, and specificity of 98.13%, 98.85%, and 97.47%, respectively [72].

The unsupervised seizure detection approach was implemented to examine the spectral information of each EEG channel individually in the alpha, theta, and delta bands, and acquired a sensitivity of 95.1% [73]. A smart headband was implemented to automatically detect seizures. The circuitry consisted of a flexible print circuit and fabric electrodes, which were integrated with a cloud computing platform. The 16 entropy features were extracted and given to the linear classifier, which effectively discriminated ictal and nonictal activity [74]. A multivariate method was applied to extract spectral graph-theoretic features to compute temporal synchronization patterns, which gave 98% sensitivity and a low latency of 6 s [75].

The enhanced transductive transfer learning Takagi–Sugeno–Kang fuzzy system was implemented and adopted WPD for feature extraction. Six features were extracted and given to the ANFIS classifier [76]. E-glass, a wearable device, was developed to give an early warning before seizure occurrence by using four scalp EEG electrodes. DWT was applied to extract nonlinear and power features that were provided to a random forest (RF) classifier to discriminate non-seizure and seizure EEG signals, which achieved a sensitivity of 93.80% and a specificity of 93.37% [77]. A shallow-dense neural network was intended to describe epilepsy by enabling global synchronization using the maximal information coefficient (MIC), which achieved accuracy, sensitivity, and specificity of 97.292%, 98.696%, and 96.116%, respectively, by adopting the shallow-dense net classifier [78]. DWT was applied to extract features, and four classifiers, such as K-NN, SVM, LDA, and artificial neural network (ANN), were adopted and provided an accuracy of 94.6% [79].

Welch’s method was used to calculate the power spectral density (PSD) from which 12 features were extracted that were nourished into two classifiers, such as the SVM and the RF classifier, to refine the precision of 94% [80]. In [81], epileptic seizures were predicted by employing deep learning approaches combined with SVM classification. In [82], the recurrent CNN was applied to long-term scalp EEG signals to detect the epileptogenic region. In [83], the baseline correction based on the median feature method was used to train and test EEG data for automatic detection of seizures.

**Figure 1:**
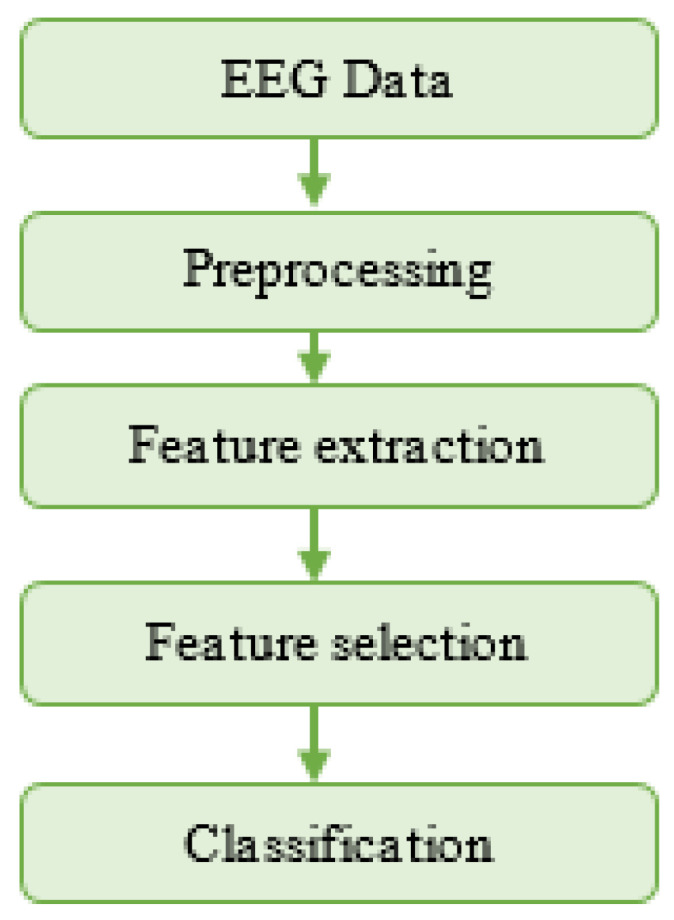
The flow chart for automatic epileptic seizure detection in artificial intelligence-based personalized medicine.

The discussed approaches can be summarized using the automatic seizure detection flow diagram shown in Figure 1, which includes the typical stages of EEG data preprocessing, feature extraction, feature selection, and classification. These stages are discussed in more detail in the following sections of this paper.

## 2. Dataset Used

**Figure 2:**
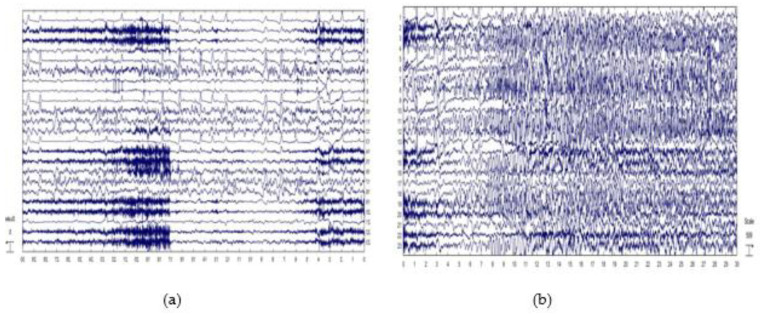
CHB-MIT database: examples of (**a**) non-seizure record; (**b**) seizure record.

The database used in this study was CHB-MIT, which is collected from the Children’s Hospital Boston. The database consists of EEG recordings with an intractable seizure of 24 pediatric patients. This database consists of 916 h of EEG records and 23 cases of EEG recordings of 22 patients whose ages ranged from 1.5 to 22 years. Continuous EEG signals were recorded after the withdrawal of antiseizure medication. The CHB-MIT database records were separated into seizure and non-seizure records and contain a total of 664 EEG files, where 198 seizures of all patients are included. These data records are one hour or four hours of data records, and 129 files contain one or more seizures, and all EEG signals were sampled at a rate of 256 samples per second with 16-bit resolution. Most EEG records contain 23 channels, and few records contain 24–26, as shown in Figure 2. The scalp EEG recording was done using the International 10–20 system. This database is available on the Physionet website. The EEG signals were segmented by the timing window since the data are long hour data.

## 3. Methods

**Figure 3:**
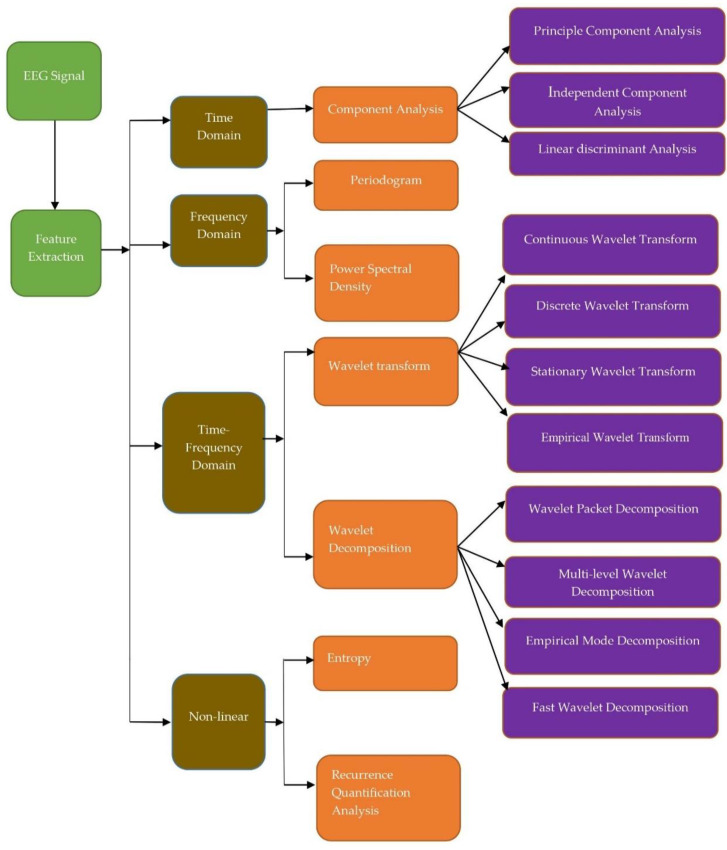
Methods to analyze EEG signals.

The EEG analysis was performed with many approaches suggested in the literature. These approaches were broadly classified into four types: (1) time domain, (2) frequency domain, (3) time-frequency domain, and (4) nonlinear methods, shown in Figure 3.

### 3.1. Time Domain

The key techniques for the analysis of the time domain were performed using component analysis and other methods to provide the discrimination between epileptic and normal patients. EEG waveforms in the time domain are associated with an epileptic and non-epileptic patient in the ictal and interictal states.

Component analysis is an unsupervised approach to extract time domain features that include PCA, independent component analysis (ICA), and LDA. The authors extracted seven features of peak frequency, median frequency, variance, root mean square (RMS), sample entropy, skewness, and kurtosis from every 115 columns, so in total, 805 features and 20 uncorrelated features were extracted by incorporating PCA and LDA [40].

PCA and common spatial patterns were defined to extract discriminative features, for example, statistical features related to a minimum, maximum, mean, variance, standard deviation, range, kurtosis, skewness, RMS, and morphological features such as curve length, zero cross, number of peaks, average nonlinear energy, and band power to provide the classification of the EEG signal [63]. Variance feature, RMS, skewness, kurtosis, peak frequency, median frequency, sample entropy, and about 20 uncorrelated features were extracted by several analyses using PCA, LDA independent search, LDA forward search, LDA backward search, and Gram–Schmidt analysis [32].

The CMIM feature selection method was used to extract features [21]. The features of skewness, kurtosis, number of maxima and minima, mean, variance, standard deviation, COV, RMS, Shannon entropy, approximate entropy, energy, standard variation, and autocovariance were extracted [22]. The time domain features of mean, standard deviation, median, skewness, kurtosis, a positive and negative value, and the first derivative of mean and max, RMS, line length were extracted [28]. Histogram-based statistical features were extracted, and by analyzing MSE, the interpolated histogram feature was extracted with ten optimal features that were collected by the COV feature, Bowley’s measure of skewness, moment measure of skewness feature, kurtosis feature, Pearson’s measure of skewness, the approximation of negative entropy feature and coefficient of IHF [42].

The statistical moments, standard deviation, zero crossing, and peak-to-peak voltage from the EEG signals were extracted to classify preictal and interictal states. Amplitude, skewness, kurtosis, and entropy features were extracted, and from the four features, amplitude and kurtosis of time domain features were selected to provide discrimination [79], as listed in Table 1.

### 3.2. Frequency Domain

Spectral and energy features were extracted from a periodogram, which was estimated by applying the Welch algorithm with 50% overlap [14]. Let *p*th windowed input signal x be represented as [12]

(1) $$x_{p}\left(n\right)≅w\left(n\right)x\left(n+pR\right),\;\;n=0,1,\ldots ,M-1,\;\;p=0,1,\ldots .,k$$

where R is the window hop size, and k is the number of the available frame. The *p*th block periodogram is given as:

(2) $$P_{x_{p}},M\left(w_{k}\right)=\frac{1}{M}{\left|{FFT}_{N,k}\left(x_{p}\right)\right|}^{2}≅\frac{1}{M}{\left|\overset{N-1}{\underset{n=0}{\sum }}x_{p}\left(n\right)e^{-\frac{j2\pi nk}{n}}\right|}^{2}$$

The Welch method of PSD is denoted by

(3) $$\mathrm{PS}\hat{D}\left(w_{k}\right)=\frac{1}{k}\left|\overset{k-1}{\underset{p=0}{\sum }}P_{x_{p,M}}\left(w_{k}\right)\right|$$

The features, such as maximum PSD, frequency of maximum PSD, mean PSD in theta, alpha, beta, gamma, and delta frequency band, and variation of PSD, were extracted from PSD evaluated by Welch’s method with a 90% overlap [79]. Spectral features were calculated with the help of PSD evaluated using the Burg method of order 16; therefore, eight spectral features were obtained [41]. The Fourier coefficient of each frequency band, which is theta, alpha, low beta, mid-beta, high beta, and gamma, was extracted by calculating PSD from an attractor in EEG [50]. Seven FIR bandpass filters were designed to extract features on 18 channels, each consisting of seven features on three-time windows so that, finally, 378 dimensions of a feature vector were formed [17].

CMIM has extracted the features of maximum, minimum, and mean power spectrum, spectral entropy, and median frequency [21]. The frequencies domain features of the maximum, minimum and mean power spectrum, spectral entropy, and median frequency were extracted [22]. Adaptive segmentation was performed, and it used the nonlinear energy operator, which segments the EEG, which was fed to the iterated filter banks to extract spectral energy features and temporal features for refined classification [30].

Feature extraction (FE) was performed by applying a two-second non-overlapped window. The feature extraction engine comprises two sets of the bandpass filter (BPF). For each channel, the frequency bands were subdivided into delta, theta, alpha, and beta, whose ranges were 0–3, 4–7, 8–15, and 16–30 Hz, respectively [43]. The FE engine consisting of seven BPF and a spectral energy calculator was used to extract features [44]. Higher-order spectral analysis was performed to extract spectral and temporal patterns [51]. The FE method was implemented in FPGA. Amplitude and frequency were extracted for seizure detection [61] as tabulated in Table 2.

### 3.3. Time-Frequency Domain

#### 3.3.1. Wavelet Transform (WT)

WT has originated as a dynamic approach in analyzing non-stationary signals. In WT, energy associated with the EEG was used to obtain wavelet coefficients [84], and it can be inferred as the filter bank [85]. It is broadly classified into CWT, DWT, and WPD. WT was utilized to extract statistical features, energy and COV features, IQR, and MAD [15]. The WT was intended to extract features from 23 channels of EEG. These features were partitioned into normal, pre-seizure, and seizure events [66]. The approximation coefficients and logarithm of variance detail coefficients were estimated to extract single wavelet-based features, which increased the precision of seizure classification [47]. The fuzzy rule-based feature extraction method was analyzed, and WT was applied to decomposition entropy of the EEG signal into sub-bands, which extract nonlinear features of the Lyapunov exponent, correlation dimension, and approximation features [64].

#### 3.3.2. Continuous Wavelet Transform (CWT)

Bivariate features were extracted by adopting CWT [16]. CWT for a signal u(t) was given as follows:

(4) $$C\left(a,b\right)=\int_{-\infty }^{+\infty }u\left(t\right)\Psi_{a,b}^{*}\left(t\right)dt$$

where a stands for the scaling, and b stands for the translation factor along the *x*-axis:

(5) $$\Psi_{a,b}\left(t\right)=\frac{1}{\sqrt{a}}\;\Psi \left(\frac{t-b}{a}\right)\;,\;a>0,\;b\in R$$

where Ψ (t) signifies the wavelet.

WT was carried out to extract temporal measures in which spectral and temporal measures where the temporal features like mean, normalized coefficient of variation (NCOV), STD, skewness, kurtosis, spectral characteristics, mean PSD, and peak PSD were extracted [49].

#### 3.3.3. Discrete Wavelet Transform (DWT)

DWT is used for the characterization of a signal as an infinite set of wavelets on an orthonormal basis [86]. DWT can decompose nonlinear structures of the signal into the approximate and the detail coefficient on the commonly used Daubechies 4 (Db4) wavelet [87]. In DWT, the translation and dilation parameters are discretized as follows:

(6) $$\{\begin{array}{c} a=a_{0}^{j}\\ b=kb_{0}a_{0}^{j} \end{array}\;a_{0}<1,\;b_{0}\;\neq 0,j\in Z,k\in Z$$

The wavelet with the parameters was assumed as

(7) $$\Psi_{j,k}\left(t\right)=\left(\frac{1}{\sqrt{a_{0}^{j}}}*\Psi \;\left(\frac{t-ka_{0}^{j}b_{0}}{a_{0}^{j}}\right)\right)$$

Therefore, DWT was given as

(8) $$D\left(j,k\right)=\int_{-\infty }^{+\infty }u\left(t\right)\Psi_{j,k}\left(t\right)\;dt$$

The signal u(t) can be reproduced using the inverse DWT as follows:

(9) $$u\left(t\right)=\frac{1}{a}\overset{J}{\underset{j=-\infty }{\sum }}\overset{\infty }{\underset{k=-\infty }{\sum }}wt\left(j,k\right)\;\Psi_{j,k}\left(t\right),\;a\in R^{+}$$

DWT was performed for five levels of wavelet decomposition to extract characteristics such as energy, NCOV, and relative coefficient of variation (RCOV) [19]. Energy, entropy, standard deviation, mean, maximum, and minimum of wavelet-based features with wavelet decomposition and statistical IQR and MAD features without wavelet decomposition were extracted to provide automatic classification of seizures [25]. DWT was applied to extract the mean, standard deviation, minimum, maximum value, median value, skewness, kurtosis, relative energy, total energy, Shannon entropy, spectral entropy, and first and second derivative of maximum and minimum values [28]. Engaging SWT to perform feature extraction where 176 frequency and 88 energy features were extracted that were mean frequency, peak frequency, relative bands energy, left anterior, right anterior, left posterior, and right posterior [41]. Multilevel wavelet decomposition was employed to extract magnitude, spectral energy variation, and relevance frequency and spectral features of maximum, minimum, and mean to provide an effective classification [46]. Wavelet-based features were extracted by engaging wavelet features from two to seven, in which the performance of each feature was obtained, and line length, nonlinear energy, variance, and maximum features were extracted for patient-dependent classification [48]. Approximation coefficients and a logarithm of variance detail coefficients were estimated to extract single wavelet-based features, enlarging the accuracy of seizure classification [58]. The entropy features were extracted from the decomposed coefficients [77]. Energy components were extracted over the delta, theta, alpha, beta, gamma1, and gamma2 frequency bands via calculating PSD by incorporating Fast Fourier Transform (FFT), and additionally, DWT was applied to extract seven-level decomposition coefficients [69]. The scattering transform and DWT were adapted to perform feature extraction and extracted 45 features related to spectra, entropies, Hurst exponent, line length, power spectra, and fractal dimensions [53].

#### 3.3.4. Wavelet Packet Decomposition (WPD)

WPD is an extension of DWT [2]. DWT decomposes the approximate coefficient, whereas WPD yields both approximate and detail coefficients [88]. In WPD, the original signal was reconstructed by combining various levels of decomposition [89]. Spectral features, fractal features, temporal features, and spatial features were extracted by performing the FWD method, also called harmonic wavelet packet transform (HWPT) [57]. Six statistical features in each sub-band were extracted by EMD, discrete wavelet transform, and wavelet packet decomposition [68]. CMIM was applied to extract the time-frequency domain features such as energy of four frequency bands, relative entropy, Shannon entropy, COV, mean, standard deviation, and frequency regularity index [21]. Short-Time Fourier Transform (STFT) was used to extract features such as relative scale energy, Shannon entropy, COV, frequency regularity index, maximum, minimum, variance, mean, standard deviation, and energy in frequency band [22]. Singular Lorenz measures approach was proposed to extract features by using SVD to estimate the singular values. Lorenz inconsistent features and Lorenz consistent were extracted, and optimal features such as Kuznets ratio, Gini coefficient, and Theil’s first ratio were also extracted. These features were obtained by IQR interpretation for the EEG signal [33].

The PARAFAC method was introduced to extract spatio-spectral features [38]. The features of correlation dimension, largest Lyapunov exponent, maximum linear cross-correlation, and nonlinear interdependence were extracted by the three steps where decomposition of EEG data was done with EMD, Multivariate EMD (MEMD), and Noise-assisted MEMD (NA-MEMD), which were given to the Hilbert transform, thereby acquiring a phase lock value for classification [52]. EWT was applied and extracted the three features where the gray-level co-occurrence matrix was used to extract multivariate textual features, and the joint features were extracted by computing the Hadamard product. The extracted features were plotted in the receiver operating characteristic (ROC) curves [54]. LGBP features were extracted by the suggested sparse rational decomposition and calculated the eight rational done with the help of rational discrete STFT. The LGBP width features and 1D LGBP features were extracted to provide discrimination of seizures and non-seizure events [55]. STFT was applied over the EEG signal, and CNN was employed to extract features for epilepsy detection [65]. STFT was applied to extract energy components in three frequency bands, namely delta, theta, and alpha, ranging 0–4, 4–7, and 8–13 Hz, respectively [70]. The summary of the time-frequency techniques is listed in Table 3.

### 3.4. Nonlinear Domain

#### 3.4.1. Recurrence Quantification Analysis (RQA)

RQA is well suited for nonlinear data analysis [90], which can capture transient states in various scenarios using EEG signals [91]. RQA was carried out to extract the RQA parameter, which is determinism, average diagonal line length, entropy, linearity, and trapping time, which was acquired from the recurrence plot [26,31]. The recurrence network was adapted to extract the RQA features, and the graph-theoretic features results were inferred from the recurrence plot [75]. The four categories of the feature extraction method of approximate entropy, sample entropy, RQA, and a wavelet-based energy-based approach [23] were adopted.

#### 3.4.2. Entropy

Entropy is a quantity of the degree of information that can be used to separate useful information from the noisy environment [92]. The uncertainty or the consistency of an EEG signal in various extents and instability variation in the signal were measured using Approximate Entropy (AE) [93,94,95]. The logarithmic probability that the signal with N sample points repeats itself within the tolerance of r for m points and for next m+1 points was expressed in approximate entropy. For a given time series y(i) of length N, N−m+1 vectors Y(1), Y(2),…,Y(N−m+1) were constructed. AE was given as follows [93]:

(10) $$ApEn\left(m,r,N\right)=\phi^{m}\left(r\right)-\phi^{m+1}\left(r\right)$$

where

(11) $$\phi^{m}\left(r\right)=\frac{1}{\left(N-m+1\right)}\underset{i}{\sum }\ln\left(C_{i}^{d}\left(r\right)\right)$$

where $C_{i}^{d}$ is a correlation integral indicating the probability of a vector Y(i), which remains similar to Y(j) within tolerance limit r.

The sample entropy was a modified version of AE. Poincare mapping was used to calculate the intersection point, which constructs a 1D sequence that extracts the seven features such as quantile, IQR, Shannon entropy, RMS, COV, energy to differentiate seizures from non-seizure records [67] listed in Table 4.

#### 3.4.3. Hjorth’s Parameters

The Hjorth’s parameters define the EEG signal in terms of its time domain features such as amplitude (activity), slope (mobility), and slope spread (complexity), thus the name “normalized slope descriptors” (NSDs) [96]. The descriptors may describe any signal in the time and frequency domains and gather its important characteristic such as energy content, frequency, and waveform complexity [97]. They have been used, among other features, to discriminate the preictal and interictal EEG in [98].

The first Hjorth’s parameter, activity, is the variance $\sigma_{X}^{2}$ of the EEG signal X.

The second Hjorth’s parameter, mobility, is expressed as:

(12) $$mobility=\frac{\sigma_{X^{\prime }}}{\sigma_{X}}$$

The third Hjorth’s parameter, complexity, is defined as:

(13) $$complexity=\frac{\sigma_{X^{\prime \prime }}/\sigma_{X^{\prime }}}{\sigma_{X^{\prime }}/\sigma_{X}}.$$

where $X^{\prime }$ is the first derivative of X obtained by differencing, while $X^{\prime \prime }$ is the first derivative of $X^{\prime }$ obtained by differencing.

### 3.5. Other Feature Extraction Methods

The nonlinear feature of the Lyapunov exponent feature was extracted [21,22]. A multivariate feature extraction approach was adopted to extract textual features, univariate, bivariate, and multivariate features extracted using channel selection; these features were mapped to the 2D image, and the GLCM matrix was applied to extract homogeneity features [29]. Mallet’s scattering transform was applied to extract Shannon entropy, Renyi entropy, permutation, and spectral entropies [53]. Eight absolute spectral power features and relative spectral power features, spectral power ratio features of 44 features were extracted by employing the sparse feature selection method, in particular, sparse Bayesian multinomial logistic regression (SBMLR), which increases classification accuracy [60]. A frequency-time division multiplexing (FTDM) filter was implemented to extract spatial, temporal, and spectral features for patient-specific seizure detection [37]. Linear and nonlinear filtering operations were applied to extract spectral-energy features from compressively sensed EEG [18]. An eight-channel feature extraction engine was developed, and the spectral, spatial, and temporal features were extracted with the help of the machine learning algorithm [20].

The stacking auto-encoders were adapted to extract discriminating features from the raw EEG [25]. Singular values, total average power, delta band average power, variance, and mean were extracted where singular and classical features were utilized for the detection of epileptic seizures, and SVD was adopted to select the singular features. The author adopted a sparse encoder to extract hidden inherent features and analyze context information to extract temporal features [39]. The machine learning algorithm was applied to extract spatial, spectral, and temporal features for EEG classification [45]. The feature used for seizure detection was the coinciding change points, which are calculated from the adaptive segmentation method [59].

The slope-based detection algorithm was developed to extract features and was also implemented in FPGA to detect seizures [62]. Spatiotemporal features were extracted to predict seizures and non-seizures by adopting 1D and 2D convolutional layers [71]. Global synchronization features were extracted by calculating the maximum information coefficient (MIC) based on a correlation matrix where seizure characteristics and non-seizure characteristics were differentiated [72]. Transductive transfer learning fuzzy systems (TTL-FS) were utilized to perform feature extraction [76]. The feature extraction method comprises three steps, namely segmentation, synchronization, and a correlation matrix based on the maximal information coefficient (CMMIC) [78].

The reconstructed phase space technique was used to create the phase space of a dynamical system represented by the EEG signal [99]. Thus, the feature vector representing the state change over time in phase space captures the system’s dynamics. The geometry of the trajectories, which can be created using a short integer or fractional time delay embedding [100], can reveal information on the EEG signal’s periodic/chaotic nature, which can be exploited for epilepsy recognition.

### 3.6. Statistical Analysis Tests

The features were analyzed using a statistical test, which was involved in the classification. The analysis of variance (ANOVA) statistical test was performed in [46,52,58,60,66], and the Mann–Whitney statistical test was carried out in [51]. The ROC curve was used to rank the features in [40,48,54,58,65,72]. The probability value (*p*-value) determined by the statistical test was used for the selection of features [51]. The p- and q-values were determined in [40], and Gram–Schmidt analysis [32] was performed.

## 4. Classification

**Figure 4:**
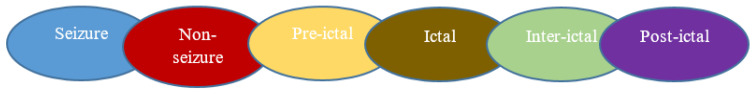
Classification stages in the diagnosis of epileptic seizures.

Classification is an essential step in the diagnosis of epileptic seizures. The stages of epilepsy in the CHB-MIT database (see Figure 4) were classified by employing various machine learning classifiers.

### 4.1. Two Class Classification (Seizure and Non-Seizure)

The authors have applied classifiers in their study to provide a better classification of epileptic seizures. Statistical features were extracted and nourished in the LDA classifier to discriminate between seizure and non-seizure classes [15]. Epileptic seizures were classified by extracting spectral energy features and employed an SVM classifier [17]. Compressed domain spectral features were extracted and given to the SVM classifier for seizure classification [18]. Energy relative values and extracted features based on NCOV were used to classify seizure and non-seizure events using the LDA classifier [19]. Spectral and spatial component features using linear SVM were involved in providing a seizure detection rate of 82.7% [20]. Time, frequency, time-frequency, and nonlinear domain features were extracted by employing seven different feature selection methods, which were classified by SVM [21]. The discrimination of seizure and non-seizure was provided by a collective network of binary classifiers using multidimensional particle swarm optimization (PSO), and the SVM classifier provides general classification where time, frequency, time-frequency, and nonlinear domain features were extracted [22]. Three nonlinear-based feature extractions were performed, and SVM and ELM were used for epileptic seizure classification [23]. Wavelet-based features and statistical features were extracted, and a linear classifier was adopted, which provided a classification accuracy of 84.2% [24]. Feature extraction was performed using the stacking autoencoder and logistic classifiers for seizure detection [25].

Entropy-based features were extracted, and these features were fed into SVM for the classification of seizures and non-seizures [27]. The time domain and frequency domain and entropy-based and discrete wavelet-based features were extracted and given into the unsupervised classification approach of k-means clustering for seizure detection [28]. The binary SVM classifier was introduced to discriminate seizure and non-seizure events [29]. The RQA features were extracted and nourished into the ECOC classifier to distinguish seizures from non-seizures [30]. Frequency domain features were extracted and given for classification of seizures and non-seizures by employing several classifiers such as LDC, Quadratic Discriminant Classifier (QDC), Uncorrelated normal density-based classifier (UDC), Polynomial classifier (POLYC), Logistic classifier (LOGLO), K-NN classifier, Decision Tree, Parzen classifier, and SVM [27]. A consistent and inconsistent measure of the extracted features was nourished into the K-NN classifier, and the final classification was provided by the MLP neural network [32]. Seven DWT nonlinear-based features were extracted and given to the two-layer classifier: the NB classifier followed by LDA. Comparative results were obtained using several classifiers, LDA, QDA, Mahalanobis discriminant analysis (MDA), NB, and SVM [34]. The SVM classifier was used for seizure detection, where classical and singular values were extracted [36]. Linear SVM was introduced to provide an epileptic seizure classification [37]. The classification between seizure and non-seizure was done by employing LDA and SVM. A comparative result was achieved by K-means clustering [38]. Hidden features and temporal features were extracted and given to SVM and a neural network (NN) for seizure classification [39]. The 805 features were extracted for the discrimination of seizures and non-seizures by adopting the K-NN classifier [40].

Spectral features were extracted and the fed into the LDA and a pattern neural network (PRNN) for the detection of seizures [41]. Histogram-based statistical features were extracted, and optimal features were selected. The MLP and Bayesian classifiers were utilized to provide better classification [42]. Frequency domain features were extracted, and linear SVM was employed to provide seizure detection [43]. Comparative classification between linear SVM and nonlinear SVM was performed where sensitivity and specificity were improved by a nonlinear SVM classifier [44]. Spectral, spatial, temporal-based features were extracted and used for classification. The best performance was achieved by RUSBoost, which was compared with RBF kernel SVM, and the proposed classifier provided performance comparable to that of the SVM [45]. Using spectral features, SVM and ELM were employed to perform classification between seizure and non-seizure events [46].

A neural network-based classifier was involved in this study based on the backpropagation algorithm for classification between seizures and non-seizures [47]. Epileptic seizure detection was performed with wavelet-based feature and time domain features employing the SVM classifier [48]. Time-frequency domain feature extraction was done, and an ELM classifier was utilized to distinguish seizures from non-seizures [49]. Three features were extracted, and classification was provided by six well-known classifiers, namely RF classifier, Functional tree (FT) classifier, K-NN, C4.5 classifier, NB, and Bayes Net [50]. LGBP features were extracted and nourished into different classifiers such as Logistic regression, random forest, and linear kernel SVM for seizure detection [55]. A genetic algorithm was utilized to provide seizure detection [56]. Energy and temporal features were extracted, and RVM was used to discriminate between seizure and non-seizure events [57]. Epileptic seizure classification was done using a slope-based detector [62]. Discrimination of seizure and non-seizure events was done by the approach of adaptive distance-based change point detector [63].

The features of the time domain and the time-frequency domain were extracted, and a fuzzy classifier was adapted to detect seizures and pre-seizure events [66]. Seven features were extracted, and two layers of classifiers involving SVM and NB classifiers for seizure and non-seizure classification were used [67]. Seizure selection methods (SSM) I, II, III, IV were introduced to classify the ictal, preictal, and interictal states [70]. Spatio-temporal features were extracted and nourished into CNN to provide a classification of seizures [71]. The VGGnet classifier was intended to provide epileptic seizure classification [72]. Spectral features were extracted, and SSM was adapted to detect seizures [73]. Epileptic seizure detection was performed with the help of the LDA classifier [74]. Time-frequency domain features were extracted, and an ANFIS classifier was employed to differentiate seizure and non-seizure events [76]. RF was used to classify seizures and non-seizure in which nonlinear features were extracted [77]. A shallow-dense net was proposed for epileptic seizure classification [78]. Statistical features were extracted, and classification was done by adopting four different classifiers ANN, K-NN, SVM, and LDA. Among these, K-NN gives better accuracy [79]. Two-class classification between seizure and normal events was performed by SVM and RF classifiers [80].

### 4.2. Classification between Ictal, Preictal, Interictal, Postictal

The SVM classifier was adopted for classification between ictal and postictal stages [14]. Bivariate features were extracted, and the SVM classifier was adapted to provide classification between the four classes, namely preictal, ictal, and interictal [16]. Statistical features were extracted, and these features were fed into the five different classifiers, namely linear SVM, logistic regression (Log-reg), K-NN, NB, and RF for preictal detection [29]. Spectral components were decomposed and fed into the SVM classifier to provide classification between interictal and preictal [52]. Spectral features were extracted and given to the kernel sparse representation classifier to classify seizures, preictal, and interictal stages [60]. Fuzzy-based features were extracted and provided in LDAG-SVM for better classification [61]. The frequency domain feature extraction was performed, and CNN was used to classify preictal and interictal EEG records [65]. The characteristics of the time-frequency domain were extracted, and discrimination of ictal and interictal and of interictal and preictal stages was provided by four different classifiers, such as SVM, RF, MLP, and K-NN [68]. Time-frequency domain features were extracted, and LSTM was used to achieve classification between the preictal and interictal states [69].

### 4.3. Classification Performance

The performance of automated classification of the EEG signal is evaluated through different performance matrices which are sensitivity, specificity, accuracy, false positive value, and positive predictive value. These matrices are mathematically given as:

(14) $$Accuracy\;\;=\frac{TP+TN}{TP+TN+FP+FN}\times 100\;$$

(15) $$Sensitivity=\frac{TP}{TP+FN}\times 100\;$$

(16) $$Specificity=\frac{TN}{TN+FP}\;\times 100\;$$

(17) $$False\;Positive\;Value=\frac{TP}{TP+FP}\times 100\;$$

(18) $$Positive\;Predictive\;Value=\frac{TP}{TP+FP}\times 100$$

where P represents the number of samples during a seizure event, and N represents the number of samples during a non-seizure event. FP (False positive) was indicated as the number of samples for a non-seizure event but erroneous for a seizure. FN (False negative) was indicated as the number of samples for a seizure event but erroneous for a non-seizure, and TP and TN are classified correctly.

Sensitivity measures the capability of the system to detect seizure events, and specificity measures the capability of the system to detect the non-seizure event. Latency is also an important metric in automated epilepsy diagnoses. Latency corresponds to the detection delay, which is the time taken by the system to detect seizures.

## 5. Conclusions

Epilepsy is a neurological disorder caused by the frequent occurrence of seizures and can be examined by EEG signals that can be useful to explore the mental states of the brain. Visual inspection and diagnosis are tedious tasks in EEG signal analysis. In this paper, various techniques that are adapted for automatic epileptic detection in the CHB-MIT dataset were presented and discussed. The feature extraction techniques in the time domain, frequency domain, time-frequency domain, and nonlinear domain were investigated. Different machine learning-based classifiers that were adapted for the classification of seizure, non-seizure, preictal, ictal, interictal, and postictal states were also discussed. The performance of each method was given in terms of sensitivity, specificity, precision, and latency, ensuring that the automatic diagnosis of epileptic seizures and their stages is highly efficient and can be implemented practically to improve the diagnosis of seizure disorders.

The summary of previous works for automated detection of epilepsy offers a perspective on the current research directions in personalized medicine towards automated seizure detection.

## Figures and Tables

**Table 1:** Summary of epileptic seizure detection approaches in the time domain.

Feature Extraction Method	Subjects	Window Size	Features	Classifier	Performance (%)
1 s non-overlapping window [21]	4 patient, 21 h recording	1 s	Time: skewness, kurtosis, No. of maxima and minima, mean, variation, standard deviation, and Shannon, entropy, ApEn, energy, standard variation, variance, and energy of auto-covariance and COV, RMS.	SVM	Sen: 90.62 Spe: 99.32
1 s non-overlapping frames [23]	21 patients (excluding patients 6, 12, 16)	1 s	Time: No. of maxima and minima, skewness, kurtosis, standard deviation, COV, RMS, Shannon entropy, ApEn, energy, standard variation, mean, variation variance, the energy of auto-covariance. Frequency: mean of the power spectrum, spectral entropy, median frequency. maximum, minimum, and Time-frequency: relative scale energy, COV, frequency regularity index, maximum, minimum, Shannon entropy, variance, mean, std-deviation, No. of extrema, and energy Nonlinear: Lyapunov exponent	SVM, multi-dimensional PSO	Sen: 89 Spe: 93
Time domain approach [28]	23 patient		Mean, std-deviation, median, skewness, kurtosis, PA value, NA_value, mean of 1st and 2nd derivative and a maximum of 1st and 2nd derivative, RMS amplitude, line length, COV	K-means clustering	
PCA [34]	23 patients excluding 15	1 s	Range, quantile, IQR, Shannon entropy, RMS amplitude, COV, and energy	LDA, NB	Sen: 88.26 Spe: 93.21
SVD [36]		1 s	Classical features such as mean, variance, kurtosis, skewness, power	SVM	Acc: 94.82
PARAFAC decomposition [38]	1 patient		Spatio-spectral features	LDA, SVM, K-means	
PCA and LDA [40]	171 seizures 171 non-seizures	60 s	Peak frequency, median frequency, variance, RMS, sample entropy, skewness, and kurtosis	k-NN classifier	Sen: 88 Spe: 88 Acc: 93
2 s non-overlapping window [43]	24 patient 198 seizures	600 s	Spectral energy features	Linear SVM, $D^{2}$ A	Sen: 95.1 Spe: 96.2
SVD [51]	23 patient	4 s	2D eigenvalues, cross bi-spectrum in the spatial and spectral direction		
PCA [62]	23 patient	1 s	Quantile, Inter quantile, range, Shannon entropy, RMS, COV, and energy	SVM NB	Sen: 95.01 Selectivity: 97.97 Acc: 96.77

**Table 2:** Summary of epileptic seizure detection approaches in the frequency domain.

Feature Extraction Method	Subjects	Window Size	Features	Classifier	Performance (%)
Welch algorithm with 50% overlap [14]	22 patients 133 seizures	5 s	Spatial and spectral	SVM	Acc: 90
Frequency band [21]	4 patients, 21 h recording	1 s	Maximum, minimum, and mean of the power spectrum, spectral entropy, median frequency.	SVM	Sen: 90.62 Spe: 99.32
Discrete Fourier Transform [28]	23 patients		Frequency: FFT_AP and RP of the delta, theta, alpha, gamma bands	K-means clustering	
Filter bank [30]	23 patients	20 s	Temporal variability information	SVM	Sen:100
PSD [32]	24 patients	60 s	Peak frequency, max frequency, median frequency, RMS, sample entropy, correlation dimension, skewness, kurtosis,	K-NN	Sen: 93 Spe: 94
IHF based [42]	23 patients, 163 seizures	30 s	Arithmetic mean, geometric mean, variance, COV, mode, median, Pearson and Bowley’s, and moment measure of skewness, kurtosis, and negative entropy	MLP, Bayesian classifier	Sen: 97.27 Acc: 86.56 Precision rate: 86.53
Attractor state analysis [47]	13 patients 143 seizures	20 s	Fourier coefficients of six EEG frequency bands		Sen: 86.67
Sparse Bayesian multinomial logistic regression [60]	17 patients 78 seizures	4 s	Spectral power and spectral power ratios such as absolute spectral power, relative spectral power, the spectral power ratio	Kernel sparse representation classifier	Sen: 86.11
STFT [70]	24 patients 198 seizures	1 s	Spectral analysis, variation in EEG energy distribution over the delta, theta, and alpha rhythms	SSM	Sen: 88
STFT [73]	24 patient 185 seizures	1 s	The energy of delta, theta, and alpha frequency bands	SSM	Sen: 95.1
Welch method with 90% overlap [80]	24 patients	20 s	Amplitude, skewness, kurtosis, entropy, maxPSD, maxF, mean Gamma, mean Beta, mean Theta, mean Delta, varPSD	SVM, RF	Acc: 94

**Table 3:** Summary of epileptic seizure detection approaches in the time-frequency domain.

Feature Extraction Method	Subjects	Window Size	Features	Classifier	Performance (%)
Wavelet decomposition [15]	24 patients 156 seizures	1 s	IQR, MAD	LDA	
CWT [16]	7 patients	5 s	Bivariate features	SVM	Sen: 52.2
Daubechies 4 wavelet transform [17]			Spectral energy	SVM	
Wavelet decomposition [19]	5 patients	1 s	COV, RCOV, NCOV,	LDA	Sen: 83.6 Spe: 100 Acc: 91.8
Wavelet decomposition [20]	23 patient	20 s	Temporal variation	Linear SVM	Acc: 82.7
DWT [21]	4 patients, 21 h recording	1 s	Time-frequency: relative scale energy, Shannon entropy, COV, frequency regularity index, maximum, minimum, variance, mean, std-deviation, No. of extrema and energy	SVM	Sen: 90.62 Spe: 99.32
Wavelet decomposition [23]	12 patients (patients 1–12)	25 s	Sample entropy, ROA features	ELM, SVM	Sen: 92.6
WT [24]	24 patients	1 s	Energy, entropy, std-deviation, maximum, minimum, mean, wavelet-based features, IQR, MAD	Linear Classifier	Sen: 98.5 Acc: 84.2
DWT [28]	23 patients		Mean, std-deviation, min, max, median, skewness, kurtosis, energy, entropy, mean and maximum of 1st and 2nd derivative, zero crossing, COV	K-means clustering	
2D mapping [29]	24 patients		Uniformity, dissimilarity, contrast, correlation, autocorrelation, sum average, variance, sum variance, entropy, sum entropy, diff entropy, diff variance, homogeneity, cluster shade, cluster prominence, max probability	SVM	Sen: 70.19 Spe: 97.74
Frequency-time division multiplexing architecture [37]	23 patients		Spectral energy	Linear SVM	Sen: 95.7 Spe: 98
SWT [41]	18 patients	2 s	Spectral and energy features 176 frequency features 88 energy features	LDA PRNN	Sen: 87.5 Spe: 99.5
Multilevel wavelet decomposition [46]	22 patients 192 seizures	10, 20, 30 min	Magnitude, spectral energy variation, and relevance frequency	SVM ELM	SVM: - Sen: 97.98 Spe: 89.90 ELM: - Sen: 99.48 Spe: 81.39
DWT [48]	24 patients	2 s	Mean, std-deviation, and all wavelet-based features	SVM	Sen: 72.99 Spe: 98.13 Acc: 96.87
Wavelet transform [49]	3 patients	2 s	Mean, normalized COV, standard deviation, skewness, kurtosis, mean DSP, Peak_PSD	ELM	Acc: 94.85
EMD, MEMD, and NA- MEMD [52]	21 patients 65 seizures	1, 5, 10, 15 s	Phase locking value	SVM	
Mallat’s scattering transform [53]	24 patients	1 s	Modulation spectra, Shannon entropy, Renyi entropy, permutation entropy, spectral entropy, Hurst exponent, line length, power spectra, fractal dimension		Spe: 86
EMD [54]	24 patients	1 s	Mean of joint instantaneous amplitude, mean monotonic absolute AM change, a variance of monotonic AM change	RF,FT, K-NN, C4.5, Bayes naïve, Bayes net	Sen: 97.91 Spe: 99.57 Acc: 99.41
FWT [57]	22 patients	2 s	Fractal dimension, correlation, wavelet coefficients, energy, and HWPT features	RVM	Sen: 96 Acc: 99.8
DWT [58]	12 patients	2 s	Wavelet-based spectral features		Sen: 83.34 Spe: 93.53 Acc: 93.24
EMD [68]	21 patients	8 s	Mean of coefficients, the average power of coefficient in every sub-band, std-deviation of coefficients, skewness, kurtosis	SVM, RF,MLP, K-NN	Sen: 99.65 Spe: 99.8 Acc: 99.7
DWT [69]	24 patients 185 seizures	5 s	Statistical moments, standard deviation, zero crossings, peak-to-peak voltage, total signal area, energy percentage at delta, theta, alpha, beta, gamma bands, cross-correlation and autocorrelation, local and global measures	LSTM	Segment based: Sen: 99.84 Spe: 99.86 Event-based: Sen: 100
WPD [76]	24 patients	10 s	Wavelet coefficients, energy features	ANFIS classifier	Sen:9 1.91 Spe: 93.16 Acc: 94.04
DWT [77]	10 patients 55 seizures	4 s	Sample, permutation, Renyi, Shannon and Tsallis entropies, and power features	RF	Sen: 93.60 Spe: 93.37
DWT [79]	10 patients	23.6 s	Std-deviation, Band power, Shannon entropy, largest Lyapunov exponent	K-NN SVM, LDA, ANN	Acc: 94.6

**Table 4:** Summary of epileptic seizure detection approaches in a nonlinear domain.

Feature Extraction Method	Subjects	Window Size	Features	Classifier	Performance (%)
Nonlinear based [21]	4 patients, 21 h recording	1 s	Lyapunov exponent	SVM	Sen: 90.62 Spe: 99.32
RQA [26]	10 seizure file		Determinism, Avg-diagonal line length, entropy, laminarity, trapping time		Sen: 97.4 Spe: 93.5
Entropy [28]	23 patients		Entropy-based: spectral, Shannon entropies	K-means clustering	
RQA [31]	10 seizure files		Determinism, Avg-diagonal line length, entropy, laminarity, trapping time	ECOC	Sen: 97.4 Spe: 93.5
RQA [75]	23 patients 182 seizures	1 s	Spatial and temporal synchronization patterns and theoretic feature		Sen: 98.48

## Data Availability

The data is available in the Physionet database at https://physionet.org/content/chbmit/1.0.0/, accessed for this review.
